# Evaluation of the Introduction of a Colorectal Bundle in Left Sided Colorectal Resections (EvaCol): Study Protocol of a Multicentre, Observational Trial

**DOI:** 10.29337/ijsp.177

**Published:** 2022-07-14

**Authors:** Benjamin Wiesler, Jörn-Markus Gass, Carsten Th. Viehl, Alexandra Müller, Jürg Metzger, Mark Hartel, Christian Nebiker, Robert Rosenberg, Raffaele Galli, Urs Zingg, Alex Ochsner, Lukas Eisner, Martina Pabst, Mathias Worni, Mark Henschel, Markus von Flüe, Markus Zuber, Marco von Strauss und Torney

**Affiliations:** 1 Clarunis University Centre for Gastrointestinal and Liver Diseases, Spitalstrasse 21, CH 4031 Basel, CH; 2 Kantonsspital Luzern, Spitalstrasse, CH 6004 Luzern, CH; 3 Spitalzentrum Biel, Vogelsang 84, CH 2501 Biel, CH; 4 Kantonsspital Aarau, Tellstrasse 25, CH 5001 Aarau, CH; 5 Kantonsspital Basel-Land, Rheinstrasse 26, CH 4410 Liestal, CH; 6 Spital Limmattal, Urdorferstrasse 100, CH 8952 Schlieren, CH; 7 Departement Chirurgie Kantonsspital Olten, Baslerstrasse 150, CH 4600 Olten, CH; 8 Klinik für Viszeral-, Gefäss- und Thoraxchirurgie Kantonsspital Olten, Solothurner Spitäler AG, Baslerstrasse 150, CH 4600 Olten, CH; 9 Stiftung Lindenhof I Campus SLB, Swiss Institute for Translational and Entrepreneurial Medicine, Freiburgstrasse 3, CH-3010 Bern, CH; 10 Head of department, Clarunis University Centre for Gastrointestinal and Liver Diseases, Spitalstrasse 21, CH 4031 Basel, CH

**Keywords:** Colorectal surgery, quality improvement measures, colorectal bundle, perioperative management, complication rate

## Abstract

**Purpose:**

Overall complication and leak rates in colorectal surgery showed only minor improvements over the last years and remain still high. While the introduction of the WHO Safer Surgery Checklist has shown a reduction of overall operative mortality and morbidity in general surgery, only minor attempts have been made to improve outcomes by standardizing perioperative processes in colorectal surgery. Nevertheless, a number of singular interventions have been found reducing postoperative complications in colorectal surgery. The aim of the present study is to combine nine of these measures to a catalogue called *colorectal bundle* (CB). This will help to standardize pre-, intra-, and post-operative processes and therefore eventually reduce complication rates after colorectal surgery.

**Methods:**

The study will be performed among nine contributing hospitals in the extended north-western part of Switzerland. In the 6-month lasting control period the patients will be treated according to the local standard of each contributing hospital. After a short implementation phase all patients will be treated according to the CB for another 6 months. Afterwards complication rates before and after the implementation of the CB will be compared.

**Discussion:**

The overall complication rate in colorectal surgery is still high. The fact that only little progress has been made in recent years underlines the relevance of the current project. It has been shown for other areas of surgery that standardization is an effective measure of reducing postoperative complication rates. We hypothesize that the combination of effective, individual components into the CB can reduce the complication rate.

**Trial registration:**

Registered in ClinicalTrials.gov on 11/03/2020; NCT04550156.

**Highlights:**

**Purpose:**

- Overall complications in colorectal surgery remain still high
- Standardizing can reduce overall operative mortality and morbidity
- Only minor attempts have been made to standardize perioperative processes in colorectal surgery
- Singular interventions have been found reducing postoperative complications
- The aim is to combine nine of these measures to a *colorectal bundle* (CB)
- The CB will help to reduce complication rates after colorectal surgery

**Methods:**

- The observational study will be performed among nine hospitals in Switzerland
- Six month the patients will be treated according to the local standards
- Afterwards patients will be treated according to the CB for another six months
- Complication rates before and after the implementation of the CB will be compared

**Discussion:**

- Only little progress has been made to reduce complication rate in colorectal surgery
- Standardization is an effective measure of reducing complication rates
- The combination of effective, individual components into the CB can reduce the complication rate

## 1. Introduction

Complications after colorectal surgery are encountered in up to 40% of patients with anastomotic leakage being the most feared potentially fatal outcome [1,2,3]. Multiple patient factors such as immunosuppression, malnutrition or severe comorbidities as well as surgeon`s and institutional factors such as caseload are known to influence the risk of complications [4,5,6]. While reported overall complication and leak rates showed only minor or no improvements over time, they differ dramatically between institutions, depending on patient`s and surgeon`s selection [5,7]. Surgical Site Infection (SSI) is a key indicator for postoperative complications in colorectal surgery. In North Western Switzerland reported anastomotic leak rates after colorectal resection range from 1.5 to 22% on an institutional level reflecting different case selection as well as surgical practice in the contributing institutions [8].

Recent research showed, that standardization is one possible measure to reduce complication rates in surgery. The introduction of the perioperative WHO Safer Surgery Checklist, for example has shown a reduction of overall operative mortality and morbidity in general surgery for high- and low-income countries [9,10]. While this has led to a widespread implementation of the checklist in most developed countries including Switzerland, only few attempts have been made to improve outcomes by standardizing the peri- and intraoperative process in colorectal surgery so far [11]. J.K. Lovely et al showed, that the implementation of an enhanced recovery pathway including preoperative analgesia, limited intravenous fluids and opiates, and early feeding, led to a significant reduction of hospitalization time in minimal invasive colorectal surgery [12]. One of the most far-reaching changes by standardization in colorectal surgery has been achieved by the introduction of the Enhanced Recovery After Surgery (ERAS) guidelines by the Society for colorectal surgery. This program, which consists of various measures for whose effectiveness there is good evidence, has had a lasting impact on the perioperative management of patients undergoing colorectal surgery [13].

Nevertheless, a number of other interventions have been found to be useful in the reduction of postoperative complications in colorectal surgery involving the following items:

1. Risk stratification of patients undergoing colorectal anastomosis can lead to a more profound and joint decision making with patients and the surgical team at what risk a particular patient will be and which patients might benefit from temporary diversion [5].
2. According to Centre for Disease Control (CDC) Guidelines for prevention of SSI patients should shower or bath (full body) with soap (antimicrobial or nonantimicrobial) or an antiseptic agent no longer than the night before surgery [14].
3. The implementation of antibiotic decontamination and mechanical bowel preparation has led to a reduction of SSI in randomized trials and a reduction of anastomotic leakage in large retrospective cohort studies [15,16].
4. As malnutrition has been identified as a key correctable risk factor in elective colorectal surgery a standardized nutritional screening should be performed among all surgical candidates. This allows for targeted nutritional support in patients with low BMI/protein levels or recent relevant loss of weight [17].
5. Adherence to WHO guidelines for intravenous antibiotic prophylaxis including proper timing within 60 minutes prior to incision as well as repetition after prolonged surgery has shown to reduce SSI rates [18,19].
6. Intraoperative anesthetic measures such as the optimization of perioperative glucose levels and securing euthermia of the patient has shown a positive effect on SSI rates [20,21,22].
7. Preparing the operative field with hair removal using clippers instead of shaving has been successfully used to reduce superficial SSI [23].
8. In a recent meta-analysis, the use of intraoperative wound protectors was associated with reduced SSI in lower gastrointestinal surgery [24]. In addition, changing gloves and instruments after the contaminated part of the procedure (opening the bowel) led to a reduction in SSI rates [25].
9. The presence of an experienced colorectal surgeon in the operation room during the formation of the anastomosis was associated with reduced complications and ostomy formation in large retrospective cohort studies [26].

All these peri- and intraoperative measures can be combined to a *colorectal bundle* (CB) to improve overall quality of care and reduce complications in colorectal surgery. To the knowledge of the authors such a comprehensive bundle has not yet been introduced.

### 1.1 Objectives

The primary objective of this study is whether the introduction of the above-mentioned quality improvement measures in a CB can reduce complication rates measured based on a reduction of the Comprehensive Complication Index (CCI) compared to the current standard [27]. We hypothesize that the introduction of the CB will lead to an overall reduction of the CCI of at least 10 points in the contributing hospitals of the Swiss North Western Hospital Network.

Secondary objectives are to

– assess the impact of the CB protocol on SSI
– assess the impact of the CB protocol on mortality
– assess the impact of the CB protocol on hospital length of stay
– assess the impact of the CB protocol on contribution margin measured as the difference between allocated costs and billed and paid income for a particular case
– assess the impact of the CB protocol on anastomotic leak rates
– identify risk factors for anastomotic leakage and higher CCI (>30) in colorectal resections
– assess the impact of the inclusion of antibiotic decontamination (facultative) into the CB in a subgroup of hospitals on CCI

## 2. Methods: Participants, interventions, and outcomes

### 2.1 Study settings

The study will be conducted in a hospital network of nine contributing hospitals in the extended north west of Switzerland. To increase generalizability, the composition of the network reaches from reference centres like the University Hospital Basel and the St. Clara Spital to middle sized cantonal hospitals. It thus covers a large health care area with a drainage area of approximately 1.5 million persons. The contributing hospitals are Clarunis University Centre for Gastrointestinal and Liver Diseases Basel with the two sites University Hospital Basel and St. Claraspital, Cantonal Hospital Baselland Liestal, Cantonal Hospital Olten, Cantonal Hospital Lucerne, Spitalzentrum Biel, Cantonal Hospital Aarau, Limmattalspital Schlieren and Lindenhofspital Bern. In the St. Claraspital 350 left sided resections are performed per year and therefore it is the biggest contributing center. Caseload of the University Hospital Basel are 120, of the Cantonal Hospital Baselland 200, of the Cantonal Hospital Olten 85, of the Cantonal Hospital Lucerne 270, of the Spitalzentrum Biel 100, of the Cantonal Hospital Aarau 85, of the Limmattalspital Schlieren 80 and of the Lindenhofspital Bern 125 left sided resections per year.

### 2.2 Eligibility criteria

#### Inclusion criteria

All patients aged ≥ 18 years undergoing any left sided emergency or elective colorectal resections for any indication with or without primary anastomosis in one of the nine participating hospitals are eligible to be part of the study population. Patients should have given or will give a general consent for the use of their data for research purposes. As this is a quality improvement project and the modified standard according to the CB is rolled out over the entire treating institution, patients cannot opt for non-treatment according to the CB but may deny data usage and acquisition within the trial by denying general consent.

#### Exclusion criteria

No general consent given for further data use and analysis. Vulnerable patients such as minors (age < 18 years) or patients with severe dementia. Index operation is a reintervention after an earlier operation within 30d (only relevant for the starting month of the trial). Unable to provide general consent & no possibility of general consent by proxy.

## 3. Interventions

### 3.1 Intervention description

During the control period, patients are treated according to their local standard of the contributing hospitals. During this period no special interventions are planned, but baseline data will be obtained. Data according to the CRF will be collected for the pre-, intra-, and postoperative course, including all complications according to the Clavien Dindo Classification [28] for each complication to calculate the primary outcome measure (CCI) [27].

After the control period the full CB is implemented during a period of 1 month in every contributing hospital.

The CB will consist of the following items:

1. Preoperative optimization of the nutritional status: Perform Nutritional Risk Score (NRS), measure albumin or prealbumin preoperatively and initiate nutritional support and delay surgery by at least two weeks if clinically feasible to allow improvement of nutritional status in case the NRS is >2.
2. Preoperative showering with an antiseptic agent on the night before or the day of admission for all patients.
3. Antibiotic decontamination (which is optional according to institutional preference) and mechanical bowel preparation (for all).
4. Administer preoperative antibiotic prophylaxis within 60 minutes prior to skin incision (not after and not any earlier).
5. Hair removal in the operative field with clippers (no shaving).
6. Peri-, intra-, and postoperative warming to maintain body temperature above 36 °C and peri-, intra- and postoperative glucose control below 9 mmol/l until patient leaves the wake-up area.
7. Usage of a wound protection device (such as Alexis® wound protector or others).
8. Change of gloves and instruments after anastomosis respectively the contaminated phase in patients with end colostomy formation.
9. Anastomosis only with experienced senior surgeon present in the operating theatre for any elective and emergency procedures (senior surgeon will be defined as follows: “Schwerpunkt Träger Viszeralchirurgie” and/or “European Board of Surgical Qualification (EBSQ) Colorectal”, and/or certified operator according to Deutsche Krebs Gesellschaft (DKG) alternatively a head of department/local principal investigator can name surgeons in case of other qualification if deemed necessary).

Once these measures are completely implemented, data collection continue for another period of 6 months including prospective monitoring of compliance to CB measures. In addition, the adherence to the ERAS program will be evaluated for every single network hospital over the entire study period, to evaluate possible synergies with the ERAS program.

### 3.2 Criteria for discontinuing interventions

The project leader is promptly notified (within 24 hours) if immediate safety and protective measures have to be taken during the conduct of the research project. The Ethics Committee will be notified of these measures and of the circumstances necessitating them within 7 days. If a serious event occurs, the research project will be interrupted and the Ethics Committee notified on the circumstances within 7 days according to Ordinance on Human Research (HRO) Art. 21.

A serious event is defined as any adverse event where it cannot be excluded, that the event is attributable to collection of health-related personal data, and which:

- requires inpatient treatment not envisaged in the protocol or extends a current hospital stay;
- results in permanent or significant incapacity or disability; or
- is life-threatening or results in death

It is important to note that since the study is purely observational, treatment related complications or morality does not fall under these regulations. Severe adverse events in the setting of this study were defined as any events related to the data collection and processing such as violations of confidentiality or others.

### 3.3 Strategies to improve adherence to interventions

Compliance to the beforehand declared local standards are monitored by chart reviews. Patients will be contacted for quality control measures 30 days after surgery to detect any post discharge complications and allow possibly necessary readmissions or outpatient controls. Adherence to the CB is monitored by checklists and overviewed by the responsible study physicians. In case of non-adherence to the protocol individual feedback is given to the responsible surgeon. The number of patients, who discontinue the interventions implemented in the colorectal bundle will be collected for every network hospital separately.

In worst case patients in centres will be excluded in case of excessive non-adherence due to possible contamination of the data pool. For this case the data collection period will be extended until full compliance to the CB is achieved for a period of at least 6 months.

### 3.4 Relevant concomitant care permitted or prohibited during the trial

For the subgroup of patients receiving antibiotic decontamination active bacterial infection requiring systemic antibiotics or patients that received IV or oral antibiotics in the past 7 days prior to the planned decontamination will lead to exclusion of this component in these particular patients. Other circumstances that lead to exclusion from antibiotic decontamination are terminal kidney disease or the impossibility to take antibiotic decontamination medication (e.g., vomiting, dysphagia) at time of screening, pregnancy or breastfeeding, emergency or expedited surgery.

### 3.5 Outcomes

The primary endpoint is the CCI at 30 days after the index procedure. The CCI is a continuous scale to measure surgical morbidity that has been developed on the basis of the Clavien Dindo Classification [28] for surgical complications and summarizes and weighs all postoperative complications to a scale from 0 (no complications) to 100 (death of the patient) [27]. This outcome allows to quantify the overall burden of complications and a more subtle detection of differences in the quality of care. It better reflects the patients’ perspective instead of solely summarizing the worst complication the patient did encounter during the course of his or her treatment.

– The first secondary endpoint is the rate of SSI at 30d.
– The second secondary endpoint is the mortality rate at 30d.
– The third secondary endpoint is the hospital length of stay.
– The fourth secondary endpoint is the contribution margin measured as the difference between allocated costs and billed and paid income for a particular case.
– The fifth secondary outcome is anastomotic leakage at 30d defined as any radiologically or clinically diagnosed anastomotic leakage.

### 3.6 Participant timeline

For participant timeline see Table 1.

**Table 1:** **a.** Schedule for elective patients. **b.** Schedule for emergency patients.


A. ELECTIVE PATIENTS					

TIME (DAYS)	>–1 DAY	–DAY1	0	+X UNTIL DISCHARGE	+30D

***Visit***	*Screening*		*OP*	*In hospital stay*	*Telephone contact* *(or review of chart/contact with treatment team in case of ongoing hospitalization)*

***oral and written general consent in outpatient clinic or at admission***	+	+	+		

***check inclusion-/exclusion criteria***	*+*				

***Record preop parameters***		*+*			

***Record intraop parameters***			*+*		

***Record postop parameters until discharge***				*+*	

***Telephone contact for post discharge complications***					*+*

**B. EMERGENCY PATIENTS**					

**TIME (DAYS)**		**0**	**1–4**	**+x until discharge**	**+30D**

***Visit***		*OP*		*In hospital stay*	*Telephone contact* *(or review of chart/contact with treatment team in case of ongoing hospitalization)*

***oral and written general consent at admission or in case of urgent cases postoperatively***		*+*	*+*		

***check inclusion-/exclusion criteria***			*+*		

***Record preop parameters***		*+*			

***Record intraop parameters***		*+*			

***Record postop parameters until discharge***				*+*	

***Telephone contact for post discharge complications***					*+*


### 3.7 Sample size

The sample size calculation described below is for an observational study. Simulations were used to estimate the required sample size. Specifically, we generated 1111 synthetic datasets for a range of potential sample sizes and possible reductions in CCI score due to the modified standard. We then applied the intended analysis (a linear mixed effects model with CCI as the outcome) to each of the simulated datasets. We modelled adoption of the modified standard as a fixed effect and the surgery performance of each study centre as a random effect to account for expected variation in outcomes.

The simulations are based on the following assumptions.

The modified standard will be studied in eight middle sized centres (Clarunis Site Universitätsspital Basel, Kantonsspital Aarau, Spitalzentrum Biel, Kantonsspital Baselland, Luzerner Kantonsspital, Spital Limmattal, Kantonsspital Olten and Lindenhofspital Bern) and one large centre (Clarunis Site St. Claraspital).

We assume that each middle size centre contributes n patients to each condition under study (before and after adoption of the CB) and that Clarunis Site St. Claraspital contributes 2 × n patients to each study condition.

The average CCI score pre-modified standard is 30 with a standard deviation of 12.5 [27].

The standard deviation for surgery outcomes among the centres under study is 20 (based on mortality data from the participating study centres provided by the Swiss Federal Office of Public Health) [8].

We assess the change in CCI using a linear mixed model with CCI after 30 days as the outcome variable, CB implementation as a binary factor and study centre as a random effect. The hypotheses are tested applying a one-sided Wald test for the regression coefficient estimated for the CB factor at the 2.5% Level and a target power of 90%.

Assuming that the CB improves CCI scores by 10 points, 5 patients are needed for each study condition from each middle-sized study centre and 10 patients for each study condition from St. Claraspital. Thus, at least 10 patients should be recruited from each small study centre and 20 patients from St. Claraspital for a combined total sample of 90 patients. This sample size allows detection of an improvement on the CCI with 90% probability (power), assuming that the CB really does reduce the CCI by 10 points on average. As the study is not randomized, the models will include all potential confounders of the true association between the intervention and the outcomes in the final models. To accurately estimate this association the sample size needs to be increased by a variance inflation factor (VIF) that reflects the correlation between the intervention and the confounding factors [29]. As this is difficult to quantify, a conservative VIF of 3.3 was applied to the estimated sample size so that a total of 149 patients per arm and a total of 298 for the study. This target sample size can be realistically achieved within the study period based on current estimated caseloads in the contributing hospitals. This power calculation was performed by the statistical department of the Clinical Trial Unit (CTU) of the Department for Clinical Research of the University Basel and reviewed by an independent statistician. The CTU is a well-established and validated unit. The secondary endpoints as described above will be analysed using descriptive statistics.

### 3.8 Recruitment

All patients that are referred for the above-mentioned emergency or elective left sided colorectal resection in the nine contributing hospitals are screened for inclusion by the lead surgeons that receive the referrals and an additional rescreening by a dedicated study nurse on a weekly basis by on the basis of the local operation room program and admission files for left sided colorectal resections that meet or did meet (in case of emergency surgery) inclusion criteria.

## 4. Methods: Data collection, management, and analysis

### 4.1 Data collection methods

For collection of the outcomes the secuTrial®data management system is used. This is a validated und international used data base. Data are extracted from the individual subject file and for each enrolled patient a CRF is generated. CRF is kept current to reflect patient status during the course of the study. All data entered into the database must also be available in the individual subject file either as worksheets or as notes taken by either the investigator or another responsible person assigned by the investigator (source documentation).

Specific training for the use of secuTrial® is provided. Plausibility rules are established to promote data quality. Furthermore, data are reviewed and verified prior to data entry completion. Data quality also is improved by regular data monitoring and planned audits by the CTU Basel. 30 days after the operation, study patients are interviewed by study nurses. Postoperative complication rates are assessed. In case of unavailability of the patients, treating physicians of the study patients are interviewed.

### 4.2 Data management

The data are transferred in the above-mentioned password protected secuTrial® database. The participant-, hospital-, and physician-identification list is stored within a server of the CTU of the University Hospital Basel separate from the patient data with access limited to the PI and two other Co-PIs. For quality assurance the Ethics Committee or an independent trial monitor may visit the research sites at any time. All involved parties keep the patient data strictly confidential. The Sponsor-Investigator is responsible to plan and conduct data monitoring. All source data and project related files and documents are accessible to the monitor and questions are discussed during the monitoring visits.

### 4.3 Statistical methods

The primary objective of the study is to be measured by the CCI 30 days post-operative compared to the CCI under standard care as the control group. The average CCI of patients treated in participating hospitals before implementation of the CB will be compared with the average CCI after implementation.

We hypothesize that the CB will be effective in reducing CCI scores 30 days post-operation as compared to standard care.

H0: The average CCI of patients treated after implementation of the CB is the same as (or higher than) the average CCI before implementation.

H1: The average CCI of patients treated after implementation of the CB is lower than the average CCI before implementation.

The analysis of the primary and secondary outcomes will be made based on a one-sided Fisher`s Exact Test with the assumption of an alpha level of p < 0.05 to be considered significant. A multivariable analysis will be performed to control for the above-mentioned confounding factors concerning the primary endpoint.

For adjusted analyses, the following potential confounding factors are accounted:

#### Patient factors

Age, sex, comorbidities (history of ischemic heart disease, cerebrovascular disease, diabetes mellitus, previous abdominal surgery, Body Mass Index (BMI) >30, smoking) nutritional status (measured as Nutritional Risk Score (NRS) [30] and serum albumin levels, use of oral anticoagulants, use of immunosuppressive medication, anaemia (measured based on preoperative haemoglobin levels), insurance class.

#### Procedural factors

Indication for surgery (malignancy (and UICC stage), inflammatory bowel disease, other), urgency (elective (planned)/expedited (within two weeks of decision to operate)/emergency (unplanned)) bowel preparation, antibiotic decontamination, primary and secondary operating surgeon (staff grade, specialization, yearly colorectal case load of responsible surgeon (lead or instructor)) and whether procedure was performed as a teaching procedure, operative approach (open, laparoscopic, robotic, converted), operative field contamination (clean-contaminated/contaminated/dirty), anastomosis formed (stapled/hand sewn/no anastomosis (end colostomy formation)/protective diversion) anastomotic configuration (side-to-side/end-to-side/end-to-end), anastomotic test performed (air leak test, endoscopy), result of test (leak/no leak), consequence of test (revision, oversewing, stoma formation).

## 5. Methods: Monitoring

### 5.1 Data monitoring

The Clinical Trial Unit (CTU) of the University Hospital Basel conducts the study-specific monitoring. The monitor periodically verifies the conduct of the study and data collection to ensure that all activities are carried out according to the protocol and that data quality and documentation in the electronic case report forms (eCRF) is accurate and complete. All source data and project related files and documents are accessible to the monitor and questions are discussed during the monitoring visits.

## 6. Ethics and dissemination

### 6.1 Protocol amendments

Substantial changes to the project set-up, the protocol and relevant project documents will be submitted to the Ethics Committee for approval according to HRO Art. 18 before implementation. Exceptions are measures that have to be taken immediately in order to protect the participants.

### 6.2 Consent or assent

General consent will be obtained according to the local hospital protocols at admission, or in case of expedited emergency admission during the course of treatment. Depending on the center, the general consent is either collected by specialized study nurses or the responsible study physicians. The Study was approved by the local ethic committee “EKNZ” (Ethikkomission Nordwest-und Zentralschweiz) on 07/09/2020 (Project-ID: 2020-01494).

### 6.3 Confidentiality

Project data is handled with uttermost discretion and is only accessible to authorized personnel who require the data to fulfil their duties within the scope of the research project. On the CRF and other project specific documents, participants are only identified by a unique participant number.

Treating institutions and physicians are reversibly anonymized within the database as well. There is no unblinded interinstitutional benchmarking. Data will not be published with identifiable information on the individual contributing hospitals or physicians.

### 6.4 Access to data

The final trial dataset will only be accessible for the PI and two co-PIs as well as the dedicated study statistician.

### 6.5 Dissemination policy

Publication of this study, data sharing or raw data access is only permitted with direct permission of the sponsor-investigator and all Co PIs. Publication of the main trial will be performed under a group authorship of all contributing hospitals (e.g. “Basel Colorectal Working Group”) and their Co PI and co-workers. Side arm projects will be performed and published based on the individual contribution to each project. Projects will be assigned within regular meetings of the PI and Co PI to individual contributing institutions. Participant level data and statistical code cannot be made accessible currently.

## 7. Discussion

The improvement of outcomes and reduction of complications in colorectal surgery is a highly relevant topic for patients and providers. A high rate of up to 40% of patients suffer from major complications after colorectal cancer surgery [7]. In these cases, surgical complications like anastomotic leakage will potentially lead to a worsened oncological outcome and lead to delay or even cancellation of adjuvant therapies [8]. The intended introduction of a CB to reduce postoperative complications has the potential to reduce these sometimes-life-threatening complications which is in the best interest of all patients undergoing colorectal surgery. The joint introduction of such quality improvement measures in an entire health care region like in the north western part of Switzerland allows a good generalizability of results from our study. There have been only few attempts to minimize the perioperative complication rate in colorectal surgery by introduction of standardized protocols that regulate the perioperative treatment. Studies of the past showed that standardization of these processes can lead to a reduction in postoperative complication rate, like it was shown for the introduction of the Enhanced Recovery After Surgery Protocol (ERAS) [31]. Some of the items from the colorectal bundle have also been included in the ERAS guidelines from 2018. These are preoperative nutritional care, administration of antibiotic prophylaxis 60 minutes before the procedure, antiseptic showering, bowel preparation and preventing of intraoperative hypothermia [13]. Other items such as changing gloves after the contaminated phase, presence of senior surgeon at formation of the anastomosis, antibiotic decontamination and shaving with clippers have not yet been included in the ERAS guidelines. Despite the parallels, the implementation of the ERAS Guidelines in the individual hospitals is sometimes variable. The simultaneous and binding introduction of a firmly defined treatment plan in an entire health region will help to uncover synergistic effects of the individual items and thus help to further scientifically substantiate the use of these items in the ERAS program.

There is another ongoing study of the European Society of Coloproctology investigating a quality control improvement program for right sided colorectal resections and anastomosis. To get a comprehensive picture of the treatment of these patients, we will use synergies to this EAGLE Trial [32].

We choose to use the CCI as the primary endpoint as it offers a modern and unique tool to measure and quantify the impact of all complications that happen to a given patient. It will best reflect their burden for patients and providers [9]. As a continuous parameter it allows to detect clinically relevant results with a lower sample size than for other purely event driven and therefore rarer endpoints such as mortality or anastomotic leakage [9]. By pooling the data from nine different institutions we are able to gain the target power within a reasonable amount of time (6 months for control and modified standard each).

## Role of sponsor

The study sponsor is involved in study design, data interpretation and decision of submitting manuscripts for publications. National and international research foundations will not have any role in study design set-up, data collection or analysis, decision to publish the study results or preparation of the study manuscript.

## References

[1] Bergvall M, et al. Better survival for patients with colon cancer operated on by specialized colorectal surgeons – a nationwide population-based study in Sweden 2007–2010. Colorectal Dis. 2019; 21(12): 1379–1386. DOI: 10.1111/codi.14760 31293019 PMC6916325

[2] Safety of primary anastomosis following emergency left sided colorectal resection: an international, multi-centre prospective audit. Colorectal Dis. 2018; 20(6): 47–57. DOI: 10.1111/codi.14373 30255647

[3] The 2017 European Society of Coloproctology (ESCP) international snapshot audit of left colon, sigmoid and rectal resections – Executive Summary. Colorectal Dis. 2018; 20(6): 13–14. DOI: 10.1111/codi.14391 30255645

[4] Borstlap WAA, et al. Anastomotic Leakage and Chronic Presacral Sinus Formation After Low Anterior Resection: Results From a Large Cross-sectional Study. Ann Surg. 2017; 266(5): 870–877. DOI: 10.1097/SLA.0000000000002429 28746154

[5] Frasson M, et al. Risk Factors for Anastomotic Leak After Colon Resection for Cancer: Multivariate Analysis and Nomogram From a Multicentric, Prospective, National Study With 3193 Patients. Ann Surg. 2015; 262(2): 321–30. DOI: 10.1097/SLA.0000000000000973 25361221

[6] Amri R, et al. Risk Stratification for Surgical Site Infections in Colon Cancer. JAMA Surg. 2017; 152(7): 686–690. 28403477 10.1001/jamasurg.2017.0505 PMC5547926

[7] Bisig B, Gutzwiller F, Domenighetti G. Incidence of operations in Switzerland related to insurance status. Swiss Surg. 1998; 4(3): 109–16; discussion 116–7. 9655004

[8] Eidgenossenschaft S. Qualitätsindikatoren der Schweizer Akutspitäler. Accessed 23.01.2020; 2016.

[9] Haynes AB, et al. A surgical safety checklist to reduce morbidity and mortality in a global population. N Engl J Med. 2009; 360(5): 491–9. DOI: 10.1056/NEJMsa0810119 19144931

[10] Haugen AS, et al. Effect of the World Health Organization checklist on patient outcomes: a stepped wedge cluster randomized controlled trial. Ann Surg. 2015; 261(5): 821–8. DOI: 10.1097/SLA.0000000000000716 24824415

[11] Tanner J, et al. Do surgical care bundles reduce the risk of surgical site infections in patients undergoing colorectal surgery? A systematic review and cohort meta-analysis of 8,515 patients. Surgery. 2015; 158(1): 66–77. DOI: 10.1016/j.surg.2015.03.009 25920911

[12] Lovely JK, et al. Case-matched series of enhanced versus standard recovery pathway in minimally invasive colorectal surgery. Br J Surg. 2012. 99(1): 120–6. DOI: 10.1002/bjs.7692 21948187

[13] Gustafsson UO, et al. Guidelines for Perioperative Care in Elective Colorectal Surgery: Enhanced Recovery After Surgery (ERAS(®) Society Recommendations: 2018. World J Surg. 2019; 43(3): 659–695. DOI: 10.1007/s00268-018-4844-y 30426190

[14] Berríos-Torres SI, et al. Centers for Disease Control and Prevention Guideline for the Prevention of Surgical Site Infection, 2017. JAMA Surg. 2017; 152(8): 784–791. DOI: 10.1001/jamasurg.2017.0904 28467526

[15] Fry DE. Review of The American Society of Colon and Rectal Surgeons Clinical Practice Guidelines for the Use of Bowel Preparation in Elective Colon and Rectal Surgery. JAMA Surg. 2020; 155(1): 80–81. DOI: 10.1001/jamasurg.2019.4551 31693066

[16] Scarborough JE, et al. Combined Mechanical and Oral Antibiotic Bowel Preparation Reduces Incisional Surgical Site Infection and Anastomotic Leak Rates After Elective Colorectal Resection: An Analysis of Colectomy-Targeted ACS NSQIP. Ann Surg. 2015; 262(2): 331–7. DOI: 10.1097/SLA.0000000000001041 26083870

[17] McDermott FD, et al. Systematic review of preoperative, intraoperative and postoperative risk factors for colorectal anastomotic leaks. Br J Surg. 2015; 102(5): 462–79. DOI: 10.1002/bjs.9697 25703524

[18] Allegranzi B, et al. New WHO recommendations on intraoperative and postoperative measures for surgical site infection prevention: an evidence-based global perspective. Lancet Infect Dis. 2016; 16(12): e288–e303. DOI: 10.1016/S1473-3099(16)30402-9 27816414

[19] Bertschi D, et al. Antimicrobial Prophylaxis Redosing Reduces Surgical Site Infection Risk in Prolonged Duration Surgery Irrespective of Its Timing. World J Surg. 2019; 43(10): 2420–2425. DOI: 10.1007/s00268-019-05075-y 31292675

[20] Hoang SC, Klipfel AA, Roth LA, Vrees M, Schechter S, Shah N. Colon and rectal surgery surgical site infection reduction bundle: to improve is to change. Am J Surg. 2019; 217(1): 40–45. *Aorn j*. 2019; 109(5): 649–654. DOI: 10.1002/aorn.12685 30025846

[21] Madrid E, et al. Active body surface warming systems for preventing complications caused by inadvertent perioperative hypothermia in adults. Cochrane Database Syst Rev. 2016; 4(4): Cd009016. DOI: 10.1002/14651858.CD009016.pub2 27098439 PMC8687605

[22] de Vries FE, et al. Meta-analysis of lower perioperative blood glucose target levels for reduction of surgical-site infection. Br J Surg. 2017; 104(2): e95–e105. DOI: 10.1002/bjs.10424 27901264

[23] Tanner J, Woodings D, Moncaster K. Preoperative hair removal to reduce surgical site infection. Cochrane Database Syst Rev. 2006: 2: Cd004122. DOI: 10.1002/14651858.CD004122.pub2 16856029

[24] Zhang L, Elsolh B, Patel SV. Wound protectors in reducing surgical site infections in lower gastrointestinal surgery: an updated meta-analysis. Surg Endosc. 2018; 32(3): 1111–1122. DOI: 10.1007/s00464-017-6012-0 29282577

[25] Junker T, et al. Prevention and control of surgical site infections: review of the Basel Cohort Study. Swiss Med Wkly. 2012; 142: w13616. DOI: 10.4414/smw.2012.13616 22949137

[26] Boyce SA, Bartolo DC, Paterson HM. Subspecialist emergency management of diverticulitis is associated with reduced mortality and fewer stomas. Colorectal Dis. 2013; 15(4): 442–7. DOI: 10.1111/codi.12022 22966859

[27] Slankamenac K, et al. The comprehensive complication index: a novel continuous scale to measure surgical morbidity. Ann Surg. 2013; 258(1): 1–7. DOI: 10.1097/SLA.0b013e318296c732 23728278

[28] Clavien PA, et al. The Clavien-Dindo classification of surgical complications: five-year experience. Ann Surg. 2009; 250(2): 187–96. DOI: 10.1097/SLA.0b013e3181b13ca2 19638912

[29] Hanley JA. Simple and multiple linear regression: sample size considerations. J Clin Epidemiol. 2016; 79: 112–119. DOI: 10.1016/j.jclinepi.2016.05.014 27393156

[30] Rasmussen HH, Holst M, Kondrup J. Measuring nutritional risk in hospitals. Clin Epidemiol. 2010; 2: 209–16. DOI: 10.2147/CLEP.S11265 21042553 PMC2964075

[31] Ripollés-Melchor J, et al. Association Between Use of Enhanced Recovery After Surgery Protocol and Postoperative Complications in Colorectal Surgery: The Postoperative Outcomes Within Enhanced Recovery After Surgery Protocol (POWER) Study. JAMA Surg. 2019; 154(8): 725–736. DOI: 10.1001/jamasurg.2019.0995 31066889 PMC6506896

[32] ESCP Safe Anastomosis ProGramme in CoLorectal SurgEry (EAGLE): Study protocol for an international cluster randomised trial of a quality improvement intervention to reduce anastomotic leak following right colectomy. Colorectal Dis. 2021; 23(10): 2761–2771. DOI: 10.1111/codi.15806 34255417

